# WAIS-IV Cognitive Profiles in Italian University Students with Dyslexia

**DOI:** 10.3390/jintelligence13080100

**Published:** 2025-08-07

**Authors:** Marika Iaia, Francesca Vizzi, Maria Diletta Carlino, Chiara Valeria Marinelli, Paola Angelelli, Marco Turi

**Affiliations:** 1 Laboratory of Applied Psychology and Intervention, Department of Experimental Medicine, College ISUFI, University of Salento, Ecotekne, Via per Monteroni s.n., 73100 Lecce, Italy; paola.angelelli@unisalento.it; 2 Laboratory of Applied Psychology and Intervention, Department of Human and Social Sciences, University of Salento, 73100 Lecce, Italy; francesca.vizzi@unisalento.it (F.V.); mariadiletta.carlino@unisalento.it (M.D.C.); 3 Cognitive and Affective Neuroscience Lab, Department of Humanities, Letters, Cultural Heritage and Educational Studies, Foggia University, Via Arpi, 155–176, 71121 Foggia, Italy; chiaravaleria.marinelli@unifg.it; 4 Department of Human and Social Studies, University of Salento, 73100 Lecce, Italy; marco.turi@unisalento.it

**Keywords:** dyslexia, adults, cognitive profile, transparent orthography

## Abstract

This study investigated the cognitive profiles of Italian university students with dyslexia using the WAIS-IV, comparing them to peers without specific learning disorders. Seventy-one participants took part: 36 with a diagnosis of dyslexia and 35 matched controls. While dyslexic adults showed lower Full Scale IQ (FSIQ) scores compared to controls, their scores remained within the average range. They showed deficits in Working Memory Index (WMI) and Processing Speed Index (PSI) but performed similarly to controls in Verbal Comprehension Index (VCI) and Perceptual Reasoning Index (PRI). Significant group differences also emerged in Arithmetic Reasoning, Symbol Search, and Coding subtests. Logistic regression identified WMI and PSI as the most reliable predictors of dyslexia, showing a good predictive value in discriminating between adults with and without dyslexia. Additionally, dyslexic adults displayed lower Cognitive Proficiency Index (CPI) scores relative to their General Ability Index (GAI), and lower FSIQ scores compared to controls. Overall, dyslexic adults exhibit a distinctive cognitive profile with strengths and weaknesses. This pattern can aid in dyslexia diagnosis, particularly in individuals who have compensated through extensive reading experience in a highly regular orthography.

## 1. Introduction

Dyslexia is a specific learning disorder characterized by persistent difficulties with accurate and fluent reading despite having adequate intelligence and appropriate educational opportunities. According to the DSM-5 (American Psychiatric Association 2013, it is the most prevalent learning disorder, affecting from 5% to 15% of the school population. Although compensation processes during development improve the reading performance of individuals with dyslexia, decoding difficulties persist and can significantly impact the academic activities of those pursuing higher education (Klassen et al. 2011; Carroll and Iles 2006). In fact, developmental dyslexia is a lifelong condition that extends into adulthood (e.g., Hatcher et al. 2002; Conti-Ramsden et al. 2009). Compared to control readers, adults with dyslexia were found to be slower in reading both words and pseudowords (particularly in terms of reading speed), as well as in spelling, naming speed, phonological processing, verbal memory, and vocabulary (for a study on Spanish, see Suárez-Coalla and Cuetos 2015; for Italian, see Vizzi et al. 2024, 2025; for a review see Reis et al. 2020). These findings further suggest that speed-based measures—more than accuracy—are particularly sensitive to reading and writing difficulties, not only in developmental stages but also in adulthood, especially in individuals learning transparent orthographies (Iaia et al. 2021; Re et al. 2011; Suárez-Coalla and Cuetos 2015; Hatcher et al. 2002). Notably, such difficulties in spelling and orthographic processing have been observed as early as childhood in Italian students with dyslexia, highlighting the early emergence and persistence of these deficits over time (Marinelli et al. 2021).

Identifying dyslexia in adults may be inherently complex, as they have already developed compensatory strategies, such as relying on their orthographic lexicon or using semantic context to overcome decoding difficulties (Miller-Shaul 2005; Corkett and Parrila 2008), resulting in varied functional profiles (AA.VV. 2022). Furthermore, in languages with consistent orthographies, years of reading practice can improve sublexical reading efficiency, which may partially offset the reading deficit (Kemp et al. 2009; Afonso et al. 2015). Notably, early sensitivity to sublexical units has been observed even in novice spellers learning in a consistent orthography (Iaia et al. 2022), suggesting that such compensatory mechanisms may begin to develop early in literacy acquisition. It is known that individuals with dyslexia who read and write in transparent orthographies tend to exhibit less severe deficits compared to those who have learned to read in more opaque orthographies (Reis et al. 2020). In highly educated Italian adults with dyslexia, an improvement in decoding accuracy has been observed, although difficulties with speed and automation persist (Martino et al. 2011). Consistently, a recent study from our research group, which employed a more experimental reading task (i.e., recording vocal reaction times), demonstrated that college students with dyslexia still show significant reading slowing across various word stimuli (Vizzi et al. 2025). Indeed, the most recent Italian guidelines for identifying learning disorders in adulthood (AA.VV. 2022) allow for a diagnosis even when reading performance falls within the normal range but at the lower limits (e.g., between −1.5 and −2 standard deviations from the mean or between the 5th and 15th percentiles), provided it is supported by clinical judgment (i.e., by anamnestic data related to educational history, family history of specific disorders, previous neuropsychological evaluations, adaptive functioning, and perceived difficulties in daily life, etc.; see also the DSM-5).

In this context, the study of cognitive profiles can further enhance the diagnostic process by providing additional evidence to support the likelihood of a dyslexia diagnosis. University students with dyslexia showed specific weaknesses in phonological processing tasks, verbal short-term memory tasks, and rapid automatized naming (Snowling et al. 1997; Hatcher et al. 2002; Nergård-Nilssen and Hulme 2014; Bogdanowicz et al. 2014). Moreover, and this is the focus of our research, adults with dyslexia tend to display a specific set of cognitive strengths and weaknesses, as highlighted by various studies (Swanson and Hsieh 2009; Reis et al. 2020; Pizzigallo et al. 2023). The Wechsler scales are the primary clinical tests for evaluating cognitive functions (Camara et al. 2000). Studies exploring the profiles of the Wechsler Adult Intelligence Scale-Third Edition (WAIS-III) in adults with dyslexia have revealed lower performance in processing speed and working memory subtests, as well as lower performance in some verbal comprehension (e.g., similarities and vocabulary) and visuo-perceptual reasoning subtests (e.g., picture completion, matrix reasoning) (Laasonen et al. 2009; Godoy de Oliveira et al. 2014). The Wechsler Adult Intelligence Scale-Fourth Edition (WAIS-IV; Wechsler 2008) provides a measure of general intellectual functioning (FSIQ) representing overall intellectual capacity. It is structured based on four composite scores measuring specific cognitive domains: Verbal Comprehension Index (VCI), Perceptual Reasoning Index (PRI), Working Memory Index (WMI), and Processing Speed Index (PSI) (Wechsler 2008). Grouping intelligence scores into these four indices has proven highly informative in the case of children (Toffalini et al. 2017; Cornoldi et al. 2019b). Pizzigallo et al. (2023), using the WAIS-IV and comparing the structure obtained from adults with Specific Learning Disorders with that of the control group, found that the g factor did not load substantially lower on the working memory factor compared to the control population, demonstrating that a four-factor structure has a good fit. However, it seems to have evident limitations, as some subtests, such as Information and especially Arithmetic, might reflect specific difficulties directly related to Specific Learning Disorders rather than general aspects of cognitive functioning. Therefore, it would be desirable and more informative to consider the individual profile of factor scores rather than relying solely on IQ scores on a real scale in this population compared to the general population (Pizzigallo et al. 2023). Consistent with research on developmental dyslexia (De Clercq-Quaegebeur et al. 2010; Wechsler 2003; Flanagan and Kaufman 2009; Brotto et al. 2017), adults with dyslexia or specific learning disorders in general, evaluated with the WAIS-IV, were found to be weaker in working memory and processing speed but better in verbal comprehension and perceptual reasoning (D’Elia et al. 2023; Pizzigallo et al. 2023; Scorza et al. 2023).

A recent study by Scorza et al. (2023) investigated the cognitive profiles of university students with dyslexia and Mixed-Type Learning Disorder using the WAIS-IV, with a focus on intraindividual variability across indices. Their findings showed that students with mixed-type learning disorders obtained significantly lower scores in working memory, processing speed, and verbal comprehension compared to both the dyslexic group and typically developing peers. These results highlight the increased cognitive vulnerability in individuals with mixed-type profiles, further supporting the relevance of a detailed analysis of index-level performance in adult SLD assessment.

The aim of this study is twofold. Firstly, it aims to assess the diagnostic utility of the most common profiles of the Wechsler Adult Intelligence Scale-Fourth Edition (WAIS-IV; Wechsler 2008). Secondly, examine the presence of discrepancies between the General Ability Index (GAI)–Cognitive Proficiency Index (CPI) and General Ability Index–Full Scale IQ (FSIQ) in the two groups, and assess whether this factor may have good predictive value in distinguishing adults with and without dyslexia. Consistent with prior research, we hypothesized that adults with dyslexia would exhibit an impaired cognitive performance profile, with the greatest deficits observed in the Working Memory Index (WMI) and Processing Speed Index (PSI). Additionally, we expected SLD adults to display a significant discrepancy in their cognitive profile, characterized by Full-Scale IQ (FSIQ) scores being lower than General Ability Index (GAI) scores compared to matched controls. The analyses conducted in this study aim to shed light on the cognitive characteristics of dyslexia in young adults, while also evaluating the effectiveness of cognitive scores and discrepancies in predicting the presence of the condition.

Although the present study adopts a strengths-and-weaknesses framework—commonly used in clinical and research practice in Italy—we acknowledge that this approach is subject to ongoing debate. Some scholars have raised concerns about its psychometric validity and diagnostic utility, suggesting that it may lack reliability in identifying learning disorders, and proposing alternative models such as low achievement or response-to-intervention frameworks (Fletcher et al. 2018; Stuebing et al. 2015). Nonetheless, the strengths-and-weaknesses model continues to be widely applied due to its ability to capture intraindividual cognitive discrepancies that may underlie specific learning difficulties, not only during developmental age (Poletti 2016; Toffalini et al. 2017), but also in adult and university populations (D’Elia et al. 2023; Pizzigallo et al. 2023; Scorza et al. 2023).

As highlighted above, this can be particularly useful in supporting the diagnosis of dyslexia when symptoms are less severe and compensation mechanisms arise from extensive reading practice and the facilitative effects of learning in a transparent orthography.

## 2. Materials and Methods

### 2.1. Participants

We assessed 71 Italian university students, consisting of 36 with dyslexia and 35 typical readers. The dyslexic group included 16 males and 20 females (mean age = 22.7 years, SD = 2.78). All participants had been diagnosed with dyslexia in accordance with the ICD-10 (World Health Organization 1992) and DSM-V criteria (American Psychiatric Association 2013), following the standard guidelines for psychodiagnostic assessment (see AA.VV. 2022). The selection criteria for inclusion in the dyslexic group were (i) a Full-Scale Intelligence Quotient (FSIQ) of 85 or higher, as measured by the Wechsler Intelligence Scale for Adults-IV (normative Italian data by Orsini and Pezzuti 2013); (ii) a reading delay (below the fifth percentile or 2 SDs the mean of the normative sample for reading speed or accuracy) on a standardized passage reading or word and pseudowords reading test (LSC-SUA; Montesano et al. 2020).

Although the Full Scale IQ (FSIQ) from the WAIS-IV was used as an inclusion criterion (≥85), subsequent analyses focused on specific index scores (e.g., Verbal Comprehension, Perceptual Reasoning, Working Memory, Processing Speed), which represent distinct cognitive constructs. Therefore, the use of WAIS-IV indices in group comparisons is methodologically independent from the use of the FSIQ as a screening threshold.

Two participants were excluded from the dyslexic group due to comorbid ADHD, based on clinical records and self-reported information. No participants were excluded based on IQ criteria, as all individuals met the FSIQ ≥ 85 threshold. None of the students in the dyslexic group had disability certificates or other psychopathological disorders, nor did they have socioeconomic or educational disadvantages, neurological or sensory deficits, or any other conditions that could explain their difficulties. The dyslexic students were compared with a control group of 35 typically developing (TD) students (25 males and 10 females; mean age = 23.2, SD = 2.46). For the control group, inclusion criteria required a Full-Scale Intelligence Quotient (FSIQ) of 85 or higher (WAIS-IV; normative Italian data by Orsini and Pezzuti), reading performance within the normal range for both speed and accuracy on both reading tests (LSC-SUA; Montesano et al. 2020), as well as the absence of a diagnosis of Specific Learning Disorders (SLDs), disabilities, or other psychopathological disorders. Control participants also had to be free from neurological or sensory deficits and not currently using medications that could influence cognitive test performance.

In line with common practice in the Italian clinical context, only individuals with a Full-Scale IQ ≥ 85 were included in the TD group (AA.VV. 2022). Although this threshold is more restrictive than those used in some international frameworks—such as the DSM-5 (American Psychiatric Association 2013)—it was adopted to ensure a clearer separation between typical and atypical cognitive functioning. In Italy, the primary diagnostic reference is the ICD-10 (World Health Organization 1992), which does not classify borderline intellectual functioning (IQ 70–85) as a distinct diagnostic category. Instead, such profiles are typically considered as specifiers within other clinical conditions.

The exclusion of individuals within the borderline range was a methodological choice aimed at reducing diagnostic ambiguity, as academic difficulties in such cases may arise from generalized cognitive limitations or reduced adaptive functioning (Vianello et al. 2017). We therefore included only participants with cognitive profiles in the average or above-average range to ensure clearer diagnostic boundaries and minimize interpretative complexity.

All the students were native Italian speakers.

All subjects were assessed at the time of enrolment in this study, ensuring that the profiles described in this study are up to date.

The dyslexic and TD groups were age-matched (*p* = 0.46) and gender-matched (*p* = 0.16), as shown in Table 1.

For each reading task, speed and accuracy were computed and converted into z-scores based on the norms provided by the battery (LSC-SUA; Montesano et al. 2020). To streamline the analysis, we created a consolidated “Composite Score” for all reading tasks by averaging the separately measured z-scores for speed and accuracy, assigning equal importance to both accuracy and speed (refer to Table 1), following the approach of Franceschini et al. (2012). Regarding reading skills investigated through LSC-SUA, statistically significant differences emerged in all types of tasks: Composite Score Word Reading (*p* < 0.0001), Composite Score Pseudoword Reading (*p* < 0.0001), and Composite Score Text Reading (*p* < 0.0001), as indicated in Table 1.

These students had voluntarily approached the university center, which provided a specific program for students with learning disorders. This study was approved by the Ethics Committee for Research in Psychology of the University (n. 71084 of 5 May 2021) and by the Data Protection Officer (DPO) of the University; the aims of this study were explained to the students, who provided written authorization for their participation in the research. This study was conducted according to the principles of the Helsinki Declaration.

### 2.2. Cognitive Profile Assessment

All participants were administered the Italian version (Orsini and Pezzuti 2013, 2015) of the fourth edition of the Wechsler Intelligence Scale for Adults (WAIS-IV; Wechsler 2008). The WAIS-IV comprises 15 subtests, 10 core and 5 supplementary, administered at the clinician’s discretion based on the individual’s characteristics and the purpose of the assessment, investigating four dimensions:

1. Verbal Comprehension (VCI):
  - Similarities: provides an estimate of verbal reasoning and concept formation, involving language development, lexical knowledge, auditory comprehension, memory, and the ability to discriminate between essential and non-essential features;
  - Vocabulary: measures lexical knowledge and formation of verbal concepts;
  - Information: assesses the ability to acquire, retain, and retrieve general information, invoking crystallized intelligence and long-term memory;
  - Comprehension (supplementary).
2. Perceptual Reasoning (PRI):
  - Block Design: measures the ability to analyze and synthesize abstract visual stimuli by capturing spatial relationships;
  - Matrix Reasoning: provides an estimate of fluid intelligence, particularly inductive reasoning, and general sequential reasoning;
  - Visual Puzzle;
  - Weight Comparison (supplementary);
  - Figure Completion (supplementary).
3. Working Memory (WMI):
  - Digit Span: offers a measure of auditory short-term memory, working memory, and attention and concentration skills;
  - Arithmetic Reasoning: estimates quantitative reasoning and attention, and concentration abilities;
  - Letter-Number Sequencing (supplementary).
4. Processing Speed (PSI):
  - Symbol Search: measures perceptual and processing speed, short-term visual memory, visuomotor coordination, cognitive flexibility, visual discrimination, and concentration ability;
  - Coding: provides a measure of processing speed, short-term memory, learning ability, visual perception, visuomotor coordination, visual scanning ability, cognitive flexibility, attention, and motivation;
  - Cancellation (supplementary).

Participants were administered only the core subtests.

In addition to the Full-Scale IQ and the four main indices, the battery also allows for the calculation of two additional indices: the General Ability Index (GAI) and the Cognitive Proficiency Index (CPI). The GAI consists of the subtests for the Verbal Comprehension Index and the Perceptual Reasoning Index, while the CPI comprises the subtests for Working Memory and Processing Speed.

### 2.3. Reading Assessment

The reading level was evaluated using the LSC-SUA reading achievement battery (Montesano et al. 2020), which includes tests for meaningful passage reading, single-word and pseudoword reading, and reading comprehension. Below is a description of the components of the reading battery.

### 2.4. Reading Assessment: Passage Reading

The passage reading test consists of a text passage titled Floripa, designed to present content with which participants are expected to have low and similar familiarity. The passage contains words of varying frequency and linguistic complexity, sentences with structures typical for adult readers, and rare or unfamiliar terms that require sublexical processing, such as “Floripa,” “Florianopolis,” “Cananvierias,” and “Lagoa” (all place names). The passage is 593 syllables long. Participants were asked to read the text aloud, and two dependent measures were recorded. Reading speed was measured in seconds per syllable, and reading accuracy was calculated as the number of errors adjusted for the amount of text read. Normative data are based on a sample of 667 students (Montesano et al. 2020).

### 2.5. Reading Assessment: Word and Pseudowords Reading

The word and pseudoword reading test involved reading aloud lists of words and pseudowords as quickly and accurately as possible. The word reading task included four lists, each containing 28 stimuli categorized as short high-frequency words, short low-frequency words, long high-frequency words, and long low-frequency words. The pseudoword reading task consisted of two lists of 28 stimuli each, derived by syllabic permutation of words from the word lists. Pseudowords were divided into short pseudowords (2–3 syllables) and long pseudowords (4 syllables).

Speed and accuracy were assessed for both word and pseudoword reading. Speed was calculated by dividing the total time taken to complete the word reading task by the 352 syllables in the test, and the total time taken to complete the pseudowords reading task by the 176 syllables in the test. Accuracy was measured by assigning an error score for each omitted or incorrectly read word or pseudoword. Normative data are based on 667 students for word reading and 666 for pseudoword reading (Montesano et al. 2020).

Pseudoword reading tasks are particularly important in the assessment of dyslexia, especially in transparent orthographies, such as Italian. Unlike real words, pseudowords cannot be identified through prior lexical knowledge and must be decoded solely via grapheme-to-phoneme conversion, thereby offering a direct measure of sublexical reading processes. In transparent orthographies, individuals with dyslexia may develop compensatory strategies that support accurate word reading yet still exhibit reduced speed—especially in pseudoword reading—revealing persistent difficulties in automatizing decoding mechanisms, even in adulthood (Suárez-Coalla and Cuetos 2015; Reis et al. 2020). For this reason, the inclusion of pseudoword reading tasks is strongly recommended in the diagnostic guidelines for adult dyslexia in Italy (AA.VV. 2022).

### 2.6. Analysis

All analyses were conducted using SPSS 27.0 and JAMOVI (version 2.6) for Windows. To compare cognitive profiles between groups, we fitted a linear mixed-effects model (LMM) with standardized scores (FSIQ, GAI, CPI) as the dependent variable. The model included fixed effects for Group (Control vs. Dyslexia), Index (FSIQ, GAI, CPI), and their interaction, with a by-participant random intercept to account for repeated measures across indices. Subsequently, two multivariate analyses of variance (MANOVA) were employed to compare the two groups across VCI, PRI, WMI, PSI, and WAIS-IV subtests, respectively. Post-hoc comparisons were conducted using Bonferroni’s method for multiple comparisons. Cohen’s d or partial eta-squared (η^2^p) was also calculated to ascertain the effect size of the differences between groups. Prior to conducting analyses on WAIS-IV scores, each dependent variable was examined for outliers, normality, and homogeneity of variances. Outliers were operationally defined as values exceeding ±3 standard deviations from the mean. Visual inspection through boxplots indicated that all data points were plausible within the context of the sample, and, thus, no observations were excluded. The assumption of normality was assessed using the Shapiro–Wilk test, and homogeneity of variance was evaluated accordingly. All assumptions were met for each variable (all *p* > 0.05). Logistic regression models were employed to examine the association between the intellectual profile and the likelihood of belonging to the dyslexia group versus the TD group, treating group membership as a binary outcome variable. Three separate logistic regression analyses were conducted. In the first model, the association of the entire WAIS-IV profile was examined, utilizing the four main indices as independent variables (VCI, PRI, WMI, PSI). Subsequently, in distinct logistic regression models, the difference between GAI–CPI and the difference between GAI–FSIQ were included as independent variables in the different models. To evaluate the efficacy of the model in discriminating between dyslexia and TD group members, the area under the curve (AUC) of the receiver operating characteristic (ROC) curve was calculated. ROC curves illustrate the relationship between sensitivity and 100 specificity, generating a curve that compares the discriminative capacity of an instrument against no discrimination. This graph provides insights into how the proportions of true positives and false positives change for various cutoff values. The area under the ROC curve (AUC) serves as a metric for evaluating the instrument’s utility, with AUC values of 1.0 indicating perfect discrimination. AUC values ≥0.80 suggest effective discrimination, while those <0.70 do not provide satisfactory discrimination (Hosmer et al. 2013). The AUC, encompassing both sensitivity and specificity, serves as a comprehensive measure describing the intrinsic validity of a test. In this context, it can be interpreted as the probability that a randomly selected affected individual is rated or ranked as more likely to be affected than a randomly chosen non-affected individual (for a comprehensive review, see Hajian-Tilaki 2013). Comparisons between AUCs were performed using the Delong test (DeLong et al. 1988). The results of the ROC curve analyses were also used to determine optimal cutoff scores. The optimal cutoff score is where the overall number of errors (i.e., false positives and false negatives) is minimized (Bewick et al. 2004). To select optimal cutoff scores, the Youden index (Youden 1950) was calculated, and the cutoff score associated with the highest J value is considered to indicate the optimal cutoff score. Graphically, J is the maximum vertical distance between the ROC curve and the diagonal line.

## 3. Results

**Figure 1:**
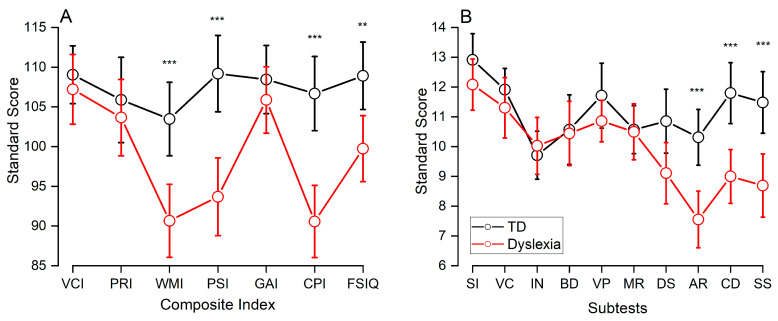
(**A**) Mean composite index scores and (**B**) WAIS-IV individual subtest scores for the dyslexia and TD groups. Note. FSIQ = Full Scale Intelligence Quotient; CPI = Cognitive Proficiency Index; GAI = General Ability Index; VCI = Verbal Comprehension Index; PRI = Perceptual Reasoning Index; WMI = Working Memory Index; PSI = Processing Speed Index; AR = Arithmetic; BD = Block Design; CD = Coding; DS = Digit Span; IN = Information; MR = Matrix Reasoning; SI = Similarities; SS = Symbol Search; VP = Visual Puzzles; VC = Vocabulary. Error bars indicate 95% confidence intervals; ** *p* < 0.001; *** *p* < 0.0001.

Table 2 and Figure 1A present group scores on the main WAIS-IV Index Scores (FSIQ, GAI, and CPI). The linear mixed-effects model assessing GAI, CPI, and FSIQ discrepancies revealed a significant main effect of Group (F_(1,69)_ = 11.4, *p* = 0.001), suggesting that the dyslexic group obtained overall lower scores than the TD group (t_(69)_ = 3.38, *p* = 0.001, d = 0.73). There was also a significant main effect of Index (F_(2,138)_ = 30.9, *p* < 0.001), with GAI scores significantly higher than both CPI (t_(138)_ = 7.72, *p* ≤ 0.001, d = 0.67) and FSIQ (t_(138)_ = 2.57, *p* < 0.001, d = 0.22) scores, and CPI scores significantly lower than FSIQ (t_(138)_ = −5.15, *p* < 0.001, d = 0.45) scores across the whole sample. Crucially, a significant Group × Index interaction (F_(2,138)_ = 18.7, *p* < 0.001) revealed distinct patterns, suggesting that the index discrepancies were not consistent across groups. To clarify this interaction, we conducted post-hoc *t*-tests (Bonferroni corrected). Our findings showed that individuals in the dyslexic group, compared to TD adults, had significantly lower CPI scores (t_(99.7)_ = 5.31, *p* < 0.001, d = 1.26) and a marginally significant difference in the FSIQ index (t_(99.7)_ = 3.02, *p* = 0.047, d = 0.72). However, no significant difference was found in GAI scores between the two groups (t_(99.7)_ = 0.84, *p* > 0.05, d = 0.20). Analysis of the performance within each group revealed no difference across Indices for the TD adults (all *p* > 0.05). However, in the dyslexic group, GAI scores were significantly higher than both CPI (t_(138)_ = 9.86, *p* < 0.001, d = 1.20 and FISQ (t_(138)_ = 3.95, *p* = 0.002, d = 0.48), and FISQ was also significantly higher than CPI (t_(138)_ = 5.95, *p* < 0.001, d = 0.72), consistent with the pattern observed in the overall sample.

Descriptive statistics for the four WAIS-IV indices for the two groups are reported in Table 3 and displayed in Figure 1A. The MANOVA on the four WAIS-IV indices yielded a significant effect of group (F_(4,66)_ = 7.84, *p* ≤ 0.0001, η^2^p = 0.32). In particular, Figure 1 suggests the presence of specific strengths and weaknesses in the cognitive profile of individuals with dyslexia compared to TD adults: indeed, planned *t*-tests (Bonferroni corrected 0.05/4 = 0.0125) revealed that the dyslexic group performed significantly lower in the WMI (t_(69)_ = 3.992, *p* < 0.001, d = 0.94) and PSI (t_(69)_ = 4.59, *p* < 0.001, d = 1.090) indices. In contrast, VCI (t_(69)_ = 0.65, *p* = 0.51, d = 0.15) and PRI (t_(69)_ = 0.62, *p* = 0.53, d = 0.14) scores were similar between the two groups.

At the level of individual WAIS-IV subtests (Table 4 and Figure 1B), we compared the dyslexic group and TD adults in a one-way MANOVA with the group as a factor. Significant results were further investigated with post-hoc *t*-tests (Bonferroni corrected). The group effect was significant (F_(10,60)_ = 4.5, *p* ≤ 0.0001, η^2^p = 0.43). Post-hoc tests showed a significant difference between groups in the following subtest: Arithmetic Reasoning (t_(69)_ = 4.19, *p* < 0.001, d = 0.99), Symbol Search (t_(69)_ = 3.82, *p* < 0.001, d = 0.90), and Coding (t_(69)_ = 4.17, *p* < 0.001, d = 0.99).

**Figure 2:**
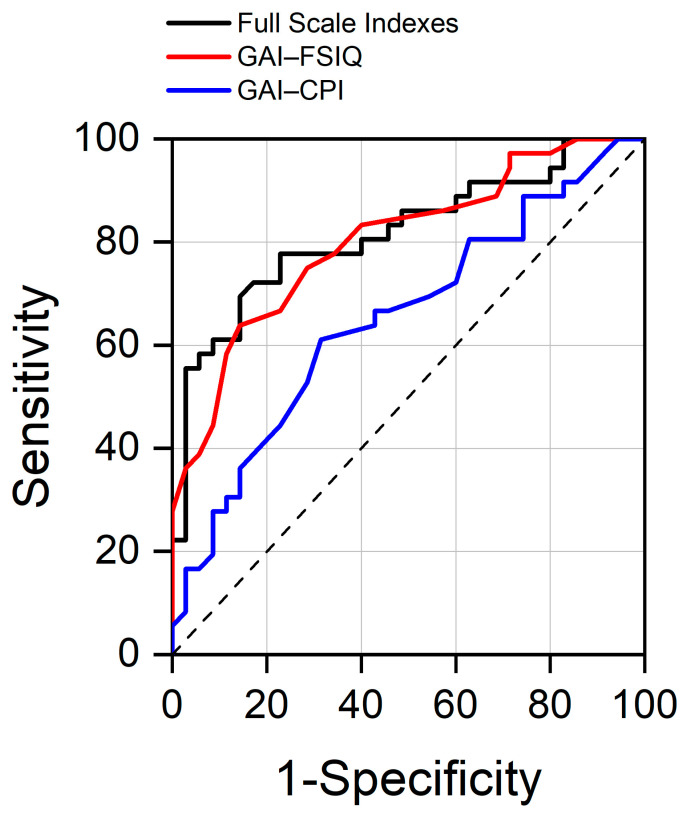
Receiver operating characteristic curve (ROC) comparing true and false positive rates among adults with Dyslexia and TD readers for the selected models: Full-Scale Indices (VCI, PRI, WMI, and PSI), GAI–CPI difference, and GAI–FSIQ difference. The dashed line is a reference line.

Three logistic regression models were applied to examine the associations between cognitive indices and group membership (dyslexia vs. TD), as presented in Table 5. The first model included the four WAIS-IV indices (VCI, PRI, WMI, and PSI) as independent variables, with the group membership (dyslexia vs. TD) as the outcome variable. In this model, only the WMI (B = −0.066, 95% CI (0.892–0.983) OR = 0.936) and PSI (B = −0.090, 95% CI (0.864–0.967) OR = 0.914) were significantly associated with group status, with an AUC of the ROC (Figure 2) curve of 0.82 (95% CI (0.718–0.918)), which is classified as “excellent discrimination”. This means that, when considering the four scale indices, a randomly selected adult with dyslexia will have lower index scores than a randomly selected adult without dyslexia approximately 82% of the time. Notably, WMI and PSI had negative coefficients, indicating that higher scores on these indices decrease the probability of dyslexia, as expected.

The second model examined the association between the GAI–CPI discrepancy score and group membership. In this model, for the GAI–CPI discrepancy (B = 0.06, 95% CI (1.006–1.117) OR = 1.06), the AUC of the ROC (Figure 2) curve was 0.65 (95% CI (0.52–0.78)), which is classified as “poor discrimination”, and the cutoff value providing the best Youden Index was 27. Consequently, the GAI > CPI cognitive score profile exhibits low diagnostic accuracy for individuals with dyslexia. The third model tested the association between the GAI–FSIQ discrepancy and group classification. In this model, for the GAI–FSIQ difference (B = 0.24, 95% CI (1.126–1.445) OR = 1.27), the AUC of the ROC (Figure 2) curve was 0.80 (95% CI (0.70–0.90)), which is classified as “excellent discrimination”, and the cutoff value providing the best Youden Index was 5. Analysis of the ROC curves showed that the model including the four WAIS-IV indices had the highest AUC. However, pairwise comparisons (De Long Test) indicated that this model’s AUC was significantly higher than that of the GAI–CPI difference model (*p* < 0.05), but not significantly different from the model including GAI–FSIQ difference (*p* = 0.75). This suggests that the model including the four indices and the one with the GAI–FSIQ difference are equivalent in the ability to detect dyslexia among participants.

## 4. Discussion

Our findings reinforce the value of cognitive assessment in identifying profiles associated with adult dyslexia, especially through WAIS-IV index scores (Wechsler Adult Intelligence Scale-Fourth Edition; Wechsler 2008; Italian validation by Orsini and Pezzuti 2013, 2015). This is particularly relevant in cases where diagnosis is complicated by compensatory mechanisms in highly educated individuals learning in transparent orthographies. In our sample, adults with dyslexia showed lower FSIQ scores than their typically developing peers, although still within the average range—a result that may seem to contrast with the traditional view of dyslexia as occurring despite adequate intelligence (e.g., American Psychiatric Association 2013). However, it is important to clarify that FSIQ, as a composite score, can be disproportionately influenced by weaknesses in working memory and processing speed, domains in which individuals with dyslexia consistently show impairments (Laasonen et al. 2009; D’Elia et al. 2023; Pizzigallo et al. 2023; Scorza et al. 2023). Consequently, the lower FSIQ observed in our sample likely reflects these known cognitive vulnerabilities rather than a global intellectual deficit. The GAI, which excludes these components, did not differ significantly between groups, an observation that supports the use of GAI as a more accurate reflection of general intellectual functioning in individuals with dyslexia (Harrison et al. 2008; Pezzuti 2016; Scorza et al. 2023). This highlights the importance of examining individual index scores and cognitive profiles, rather than relying solely on FSIQ, when assessing adults with dyslexia. Furthermore, current diagnostic frameworks recognize that dyslexia can occur across the full spectrum of intelligence, and that reading impairments may persist even in highly educated and well-compensated individuals (Reis et al. 2020; Snowling et al. 1997). Indeed, the analysis of scores obtained in the four indices revealed the presence of certain strengths and weaknesses in the cognitive profile of participants with dyslexia compared to TD adults. Specifically, individuals with dyslexia scored significantly lower on the WMI and PSI indices, while VCI and PRI were similar between the two groups. These findings align with the existing literature on adults with SLD using WAIS (version III or IV), suggesting that adults with SLD present a profile with VCI and PRI higher than WMI and PSI (Pizzigallo et al. 2023; D’Elia et al. 2023; Scorza et al. 2023; Longman 2004; Laasonen et al. 2009).

At the level of individual subtests comprising the WAIS-IV, significant differences were found between groups in the Arithmetic Reasoning subtest (under WM Index), Symbol Search, and Coding (under PS Index). The dyslexic group exhibited poor performance in the Coding subtest, as reported in other studies (Bennett et al. 2008; Brunswick et al. 2010; Godoy de Oliveira et al. 2014; Laasonen et al. 2009; Pizzigallo et al. 2023), as well as in Symbol Search (Pizzigallo et al. 2023; Gontkovsky and Golden 2022). These results indicate a generalized processing speed difficulty, already documented in children with dyslexia (for a meta-analysis, see Zoccolotti et al. 2018) and explained by theoretical models such as the Rate and Amount Model (Faust et al. 1999) and the Difference Engine Model (Myerson et al. 2003). This deficit, which stems from the interaction between cognitive speed and task complexity, has also been observed in adults with dyslexia (Vizzi et al. 2025), although it does not consistently appear across all cognitive tasks. These findings underscore the clinical relevance of processing speed deficits in dyslexia across the lifespan.

Additionally, adults with dyslexia performed poorly on the working memory subtest of Arithmetic Reasoning, reflecting specific difficulties related to dyslexia rather than general cognitive functioning. Similar results were reported by Pizzigallo et al. (2023), who noted that Arithmetic Reasoning engages both working memory and fluid reasoning, and partially reflects school achievement, making it inadequate for measuring any single factor in individuals with learning disorders. The indices GAI and CPI were designed to provide two different views of a subject’s cognitive abilities, especially when there is significant and rare variability among the four indices (VCI, PRI, WMI, PSI). Therefore, we evaluated their association with group classification, and both WMI and PSI emerged as significant predictors.

This suggests that considering the four scale indices, a randomly selected dyslexic adult will have lower indices than a randomly selected non-dyslexic adult about 82% of the time. Hence, a discrepancy between the VCI and PRI indices (constituting GAI) and the other two (constituting CPI) seems to have diagnostic power, consistent with findings in developmental age (Cornoldi et al. 2019a; Giofrè et al. 2017). Our study revealed that individuals in the dyslexic group had lower CPI scores compared to the GAI and FISQ scores and compared to the TD group. Moreover, the within-group analysis showed no difference between the indices for the TD group, while, for the dyslexic group, GAI scores were significantly higher than CPI and FSIQ, and the latter was also higher than CPI, consistent with the overall sample findings. These results align with other studies comparing GAI and total IQ in adults with specific learning disorders, showing that they had lower CPI than GAI, and the latter was always equal to or higher than the total IQ (Harrison et al. 2008; Longman 2004; Scorza et al. 2023).

In individuals with dyslexia, it is common to observe relatively preserved reasoning abilities alongside weaknesses in working memory and processing speed. Therefore, a GAI > CPI discrepancy reflects this cognitive dissociation—a profile characterized by strong reasoning in the presence of reduced cognitive efficiency (Cornoldi et al. 2019a).

The GAI aggregates scores from the Verbal Comprehension Index (VCI) and Perceptual Reasoning Index (PRI), which are less influenced by attentional and processing speed constraints. In contrast, the CPI reflects more efficiency-based functions, combining Working Memory Index (WMI) and Processing Speed Index (PSI) scores. As such, a significant discrepancy between GAI and CPI suggests that, while an individual may have intact or even above-average reasoning skills, their working memory and processing speed are comparatively weaker, cognitive characteristics commonly observed in individuals with dyslexia.

On the other hand, the GAI–FSIQ discrepancy conceptually captures the extent to which cognitive proficiency weaknesses (in working memory and processing speed) lower the overall intelligence quotient. Since the FSIQ includes all four indices, it may underestimate general intellectual potential when cognitive efficiency is compromised, particularly in adults with dyslexia, who often show relatively preserved reasoning skills despite persistent difficulties in these domains. In this sense, a GAI > FSIQ discrepancy may reflect the dissociation between intellectual potential and cognitive efficiency, which is clinically relevant in compensated or borderline cases, especially in highly educated populations using transparent orthographies, such as Italian populations.

Importantly, while the GAI–FSIQ discrepancy showed strong discriminative utility in our sample, it is essential to note that difference scores derived from highly correlated indices—such as GAI and FSIQ—may present psychometric limitations, including reduced reliability (see Stuebing et al. 2015; Fletcher et al. 2018). Based on normative data from the Italian WAIS-IV (Orsini and Pezzuti 2013; Pezzuti 2016), the reliability of the FSIQ–GAI difference score was estimated to be 0.38. This low level of reliability suggests that the observed discrepancy is not a stable individual measure and is likely to be substantially influenced by measurement error.

Consequently, the FSIQ–GAI discrepancy should not be used as a standalone diagnostic tool, and any clinical interpretation should be made only within the context of a broader, multi-source assessment framework. Furthermore, similar cognitive discrepancies (e.g., GAI > FSIQ or GAI > CPI) may occur in other neurodevelopmental conditions, such as ADHD and Autism Spectrum Disorder (ASD), thereby reducing their diagnostic specificity for dyslexia alone (Harrison et al. 2008; Gontkovsky and Golden 2022).

While previous studies (e.g., Giofrè et al. 2017) have reported moderate discriminative capacity for the GAI–FSIQ discrepancy in children with learning disorders, the psychometric limitations we outline here argue against its use as a reliable marker at the individual level.

Therefore, we recommend interpreting the GAI–FSIQ difference as a supplementary observation rather than a diagnostic indicator. Greater weight should be given to the GAI–CPI discrepancy, which, although less strongly associated with group classification in our sample, may still offer valuable information, and to the analysis of individual index scores, particularly WMI and PSI. These latter indices demonstrated stronger group-level differentiation in our study and are supported by both clinical theory and more robust psychometric properties.

In conclusion, our results suggest that the cognitive profile of Italian college students with dyslexia is more differentiated, showing both strengths and weaknesses, compared to typically developing students (see Masutto and Cornoldi 1992; Cornoldi et al. 2019a). This profile may assist in diagnosing dyslexia in cases of borderline reading performance, such as those that might be observed in highly educated adults who read and write in transparent orthographies. This study has several limitations that should be considered when interpreting the results. A primary limitation is the relatively small sample size used in the logistic regression analyses. Although the models yielded promising discriminative performance—particularly for the four-index model and the GAI–FSIQ discrepancy—the limited number of participants may have affected the reliability and generalizability of these findings. Small samples are more susceptible to overfitting, which can lead to an overestimation of model accuracy (e.g., AUC) and instability of parameter estimates. These constraints should be considered when interpreting the diagnostic utility of the WAIS-IV cognitive indices in adult dyslexia. Future studies with larger samples are necessary to replicate and validate these preliminary findings and to better characterize the potential of cognitive discrepancy measures as diagnostic tools in clinical practice.

Furthermore, the use of IQ-based discrepancy scores, such as GAI–FSIQ and GAI–CPI, while informative, involves indices that are derived from highly correlated scales and may suffer from reduced psychometric reliability. Additionally, in contexts such as the present study—where IQ ≥ 85 is already used as an inclusion criterion—there is a potential for redundancy or circularity when interpreting subsequent IQ-based differences. Future research should explore alternative statistical or data-driven approaches that can reduce these biases and improve interpretive clarity.

Additionally, the dyslexic group included a significant number of late-diagnosed students who likely missed access to early protective factors, such as reinforcement programs aimed at improving reading and writing skills, as well as sufficient social support from family and school environments. These factors could have influenced the development and evolution of specific aspects of their cognitive profiles. Research indicates that college students with specific learning disorders often experience more pronounced psychological challenges compared to typically developing young adults, stemming from the ongoing experience of their deficiencies (Iaia et al. 2024; Carpinelli et al. 2021). Additionally, the exclusive focus on university students introduces a potential selection bias, as this population represents a subset of individuals with dyslexia who are likely more academically successful, motivated, and compensated than those who do not pursue higher education. This may limit the applicability of the findings to individuals with less formal education or differing levels of academic achievement. Overall, these findings should be considered preliminary. Future studies with larger, more heterogeneous samples—including participants from diverse educational and socioeconomic backgrounds—along with meta-analytic work, will be needed to validate and extend the current results. Additionally, future research should address these limitations by examining the influence of early support and intervention on cognitive outcomes.

## 5. Conclusions

The analysis of the performance of young adults with dyslexia on the WAIS-IV has significant clinical implications as it can contribute to the diagnostic discussion and guide clinical practice. However, it is important to note that, while the WAIS-IV provides valuable insights, it should not be used in isolation for diagnosis. Other factors, such as the individual’s background, personal history, and external challenges, should also be considered. Diagnosing Specific Learning Disorders (SLDs) in adulthood is often complex, as many individuals develop compensatory strategies that mask overt difficulties. Consequently, the absence of clear symptoms does not exclude the presence of a learning disorder. Subtle difficulties may still affect daily functioning, academic performance, and emotional–motivational aspects, even if this is not immediately visible. Analyzing the scores provided by the WAIS-IV can further help the clinician understand the intellectual functioning of the young adult, identify strengths and weaknesses in cognition, and identify patterns that could support a diagnosis of dyslexia or other Specific Learning Disorders. This analysis should be seen as part of a comprehensive evaluation that includes multiple sources of information rather than a definitive answer. In light of our findings, multicomponent cognitive assessment proves essential not only for confirming or excluding a diagnosis but also for defining an individual’s functioning profile. This, in turn, supports the development of tailored strategies that promote both academic success and personal well-being. Ultimately, the goal is to provide personalized support that enhances quality of life by recognizing both cognitive resources and vulnerabilities.

## Figures and Tables

**Table 1:** Demographic data and reading skills scores (z-scores) assessed by LSC-SUA for dyslexic and typically developing (TD) adults: comparison between the two groups.

	Dyslexia Group (36) M ± SD (Range)	Control Group (35) M ± SD (Range)	Group Comparisons	*p*	Effect Size
Age, years	22.72 ± 2.78 (18–33)	23.21 ± 2.46 (19–31)	t_(69)_ = 0.74	0.464	d = 0.17
Gender (M/F)	20/16	25/10	χ^2^ = 1.93	0.165	Phi = 0.17
Composite Score Word Reading	−3.32 ± 2.22 (−9.68–−0.28)	−0.04 ± 1.12 (−2.90–2.06)	t_(69)_ = 7.82	<0.0001	d = 1.86
Composite Score Pseudoword Reading	−3.20 ± 1.89 (−7.45–−0.43)	−0.10 ± 1.08 (−3.26–1.95)	t_(69)_ = 9.01	<0.0001	d = 2.14
Composite Score Text Reading	−5.81 ± 3.05 (−12.6–0.04)	−0.63 ± 1.41 (−4.35–1.29)	t_(69)_ = 9.13	<0.0001	d = 2.17

**Table 2:** Descriptive statistics (mean standard score, SD (standard deviation), range) and linear mixed model results for group differences on the main WAIS-IV Index Scores. Significant results are highlighted in bold. Post-hoc comparisons were conducted using Bonferroni’s method for multiple comparisons, and effect size was evaluated with Cohen’s d.

WAIS-IV Indices	Dyslexia Group (N = 36) M ± SD (Range)	TD Group (N = 35) M ± SD (Range)	Group Comparisons	*p*	Effect Size
Full-Scale IQ	99.75 ± 12.27 (80–133)	108.91 ± 12.34 (85–129)	t_(99.7)_ = 3.02	**0.047**	0.72
General Ability Index	105.89 ± 12.35 (81–136)	108.46 ± 12.5 (86–133)	t_(99.7)_ = 0.84	>0.05	0.20
Cognitive Proficiency Index	90.58 ± 13.41 (70–120)	106.69 ± 13.61 (83–140)	t_(99.7)_ = 5.31	**<0.0001**	1.26

**Table 3:** Descriptive statistics (mean standard score, SD (standard deviation), range) and univariate ANOVA results for group differences in WAIS-IV Index Score. Significant results are highlighted in bold. Bonferroni correction was applied (Bonferroni Corrected alpha = 0.05/4), and effect size was evaluated with Cohen’s d.

WAIS-IV Indices	Dyslexia Group (N = 36) M ± SD (Range)	TD Group (N = 35) M ± SD (Range)	Group Comparisons	*p*	Effect Size
Verbal Comprehension	107.22 ± 12.96 (84–139)	109.06 ± 10.58 (90–127)	F_(1,69)_ = 0.426	0.516	d = 0.15
Perceptual Reasoning	103.67 ± 14.19 (71–131)	05.89 ± 15.65 (77–139)	F_(1,69)_ = 0.392	0.533	d = 0.14
Working Memory	90.67 ± 13.61 (69–123)	103.49 ± 13.44 (77–134)	F_(1,69)_ = 15.934	**<0.0001**	d = 0.94
Processing Speed	93.69 ± 14.44 (58–126)	109.20 ± 14.00 (81–150)	F_(1,69)_ = 21.08	**<0.0001**	d = 1.090

**Table 4:** Descriptive statistics (mean standard score, SD (standard deviation), range) and univariate ANOVA results for group differences in WAIS-IV subtest scores. Significant results are highlighted in bold (Bonferroni corrected alpha = 0.05/10), and effect size was evaluated with Cohen’s d.

WAIS-IV Subtests	Dyslexia Group (N = 36) M ± SD Range	TD Group (N = 35) M ± SD Range	Group Comparisons	*p*	Effect Size
Similarities	12.08 ± 2.53 (5–18)	12.91 ± 2.56 (7–17)	F_(1,69)_ = 1.889	0.174	d = 0.32
Vocabulary	11.31 ± 2.99 (7–18)	11.91 ± 2.07 (7–15)	F_(1,69)_ = 0.986	0.324	d = 0.23
Information	10.03 ± 2.82 (6–16)	9.71 ± 2.34 (6–15)	F_(1,69)_ = 0.258	0.613	d = 0.12
Block Design	10.44 ± 3.18 (4–15)	10.57 ± 3.40 (4–17)	F_(1,69)_ = 0.26	0.872	d = 0.03
Visual Puzzles	10.86 ± 2.05 (6–15)	11.71 ± 3.16 (7–19)	F_(1,69)_ = 1.82	0.182	d = 0.32
Matrix Reasoning	10.50 ± 2.78 (6–18)	10.57 ± 2.34 (7–16)	F_(1,69)_ = 0.014	0.907	d = 0.02
Digit Span	9.11 ± 3.04 (3–16)	10.86 ± 3.12 (5–17)	F_(1,69)_ = 5.592	0.02	d = 0.56
Arithmetic Reasoning	7.56 ± 2.81 (2–15)	10.31 ± 2.73 (5–16)	F_(1,69)_ = 17.57	**<0.0001**	d = 0.99
Coding	9.00 ± 2.67 (4–15)	11.80 ± 2.97 (6–19)	F_(1,69)_ = 17.404	**<0.0001**	d = 0.99
Symbol Search	8.69 ± 3.13 (1–17)	11.49 ± 3.01 (7–19)	F_(1,69)_ = 14.624	**<0.0001**	d = 0.90

**Table 5:** Ability of WAIS-IV indices and discrepancy scores to distinguish adults with dyslexia from TD adults. AUC: area of receiver operating characteristic curve; CI: confidence interval; Four scale Indices (VCI, PRI, WMI, and PSI); GAI–FSIQ (Discrepancy Score between General Ability Index and Full Scale IQ); GAI–CPI (Discrepancy Score between General Ability Index and Cognitive Proficiency Index). De Long Pairwise Comparison Differences Between AUCs. * indicate significant pairwise comparisons between AUC. * *p* < 0.05.

Models	Specificity	Sensitivity	Youndex Index	AUC (95% CI)	De Long Test
Four scale Indices	0.77	0.77	0.549	0.82 (0.71–0.91)	a > c *
GAI–FSIQ	0.85	0.63	0.496	0.80 (0.70–0.90)	b > c *
GAI–CPI	0.68	0.61	0.297	0.65 (0.52–0.78)	a > c *; b > c *

## Data Availability

The data presented in this study are available on request from the corresponding author. The data are not publicly available due to privacy and ethical restrictions, as they contain sensitive information.
